# Oxidative Balance Score and New-Onset Type 2 Diabetes Mellitus in Korean Adults without Non-Alcoholic Fatty Liver Disease: Korean Genome and Epidemiology Study-Health Examinees (KoGES-HEXA) Cohort

**DOI:** 10.3390/antiox13010107

**Published:** 2024-01-16

**Authors:** Mid-Eum Moon, Dong Hyuk Jung, Seok-Jae Heo, Byoungjin Park, Yong Jae Lee

**Affiliations:** 1 Department of Family Medicine, Yonsei University College of Medicine, Seoul 03722, Republic of Korea; eliya85@yuhs.ac (M.-E.M.); balsan2@yuhs.ac (D.H.J.); bjpark96@yuhs.ac (B.P.); 2 Department of Family Medicine, Gangnam Severance Hospital, Seoul 06273, Republic of Korea; 3 Department of Family Medicine, Yongin Severance Hospital, Yongin 16995, Republic of Korea; 4 Division of Biostatistics, Department of Biomedical Systems Informatics, Yonsei University College of Medicine, Seoul 03722, Republic of Korea; sjheo@yuhs.ac

**Keywords:** oxidative balance score, type 2 diabetes, NALFD, KoGES-HEXA cohort

## Abstract

The oxidative balance score (OBS) is a novel composite of pro- and anti-oxidative markers for assessing the risk of cardiometabolic diseases and non-alcoholic fatty liver disease (NAFLD). However, it has not yet been established whether the OBS is related to type 2 diabetes mellitus (T2DM), especially in a population without NALFD. Therefore, we aimed to investigate the longitudinal effect of the OBS on T2DM in a large cohort of Korean adults without NALFD. Data were assessed from 9798 participants without NALFD from the Korean Genome and Epidemiology Study-Health Examinees (KoGES-HEXA) cohort. The participants were divided into three groups according to OBS tertiles, identified as T1–T3. We prospectively assessed the hazard ratios (HRs) with 95% confidence intervals (CIs) for new-onset T2DM using multivariable Cox proportional hazard regression models over 6 years following the baseline survey. During the mean 3.5 years of follow-up, 145 individuals (1.48%; 56 men and 89 women) developed T2DM. The HRs of T2DM for the OBS tertiles were 0.79 (95% CI, 0.53–1.18) and 0.60 (95% CI, 0.39–0.93) in the T2 and T3 groups after adjusting for metabolic parameters in subjects without NALFD, respectively; however, the T2 group did not show statistical significance toward a decrease in incident T2DM. A low OBS may be a useful predictive marker in new-onset T2DM for middle-aged and older subjects without NALFD. This implies that the OBS could be an additional valuable tool for assessing the incidence of T2DM among individuals without NAFLD.

## 1. Introduction

According to the International Diabetes Federation, since the first report was published in 2000, the prevalence of diabetes in adults aged 20–79 has more than tripled, from approximately 151 million (4.6% of the global population at the time) to 536.6 million (10.5%) as of 2021 [1]. South Korea is no exception, with an estimated 44.3% of adults aged 30 and over in 2020 having prediabetes, and the prevalence of diabetes reaching 16.7% [2]. Poorly controlled diabetes increases the risk of co-morbidities and all-cause mortality, including cardiovascular, cerebrovascular, and peripheral vascular diseases, in the long term [3]. Therefore, early identification and prevention of risk factors for diabetes is an important public health challenge [3].

Oxidative stress is defined as an imbalance between reactive oxygen species (ROS) production and antioxidant defenses from endogenous and exogenous sources [4]. The oxidative balance score (OBS) is a composite metric that includes dietary and lifestyle factors, with a higher OBS indicating that antioxidants predominate over oxidants. To date, there is considerable evidence that a higher OBS is negatively associated with the incidence of various metabolic diseases, including new-onset hypertension [5], non-alcoholic fatty liver disease (NAFLD) [6], and chronic kidney disease (CKD) [7]. There is also evidence that a higher OBS in the general population is associated with a lower incidence of T2DM [8]. In some cross-sectional studies, the OBS was inversely related to the prevalence of T2DM and glycemic control among T2DM patients [9,10], and a recent prospective cohort study reported the protective effect of the OBS on T2DM incidence in the general population [8].

However, most T2DM patients also have other metabolic diseases, and there are numerous interactions between their pathogeneses. Among them, the strong bidirectional relationship between T2DM and NAFLD is well known [11,12], and in fact, it is estimated that more than two-thirds of T2DM patients also have NAFLD [13]. Therefore, NAFLD can interfere with the positive impact of lifestyle modifications on T2DM incidence, as represented by the OBS. Therefore, to fully understand their interdependence [14], it is necessary to determine the role of the OBS in the development of T2DM in populations for which NAFLD is excluded [15]. To our knowledge, no study has identified strong evidence of an epidemiologically inverse relationship between the OBS and T2DM risk in a prospective cohort without NAFLD. Therefore, we investigated the effect of the OBS on the incidence of T2DM in middle-aged and older adults without NAFLD through a large community-based prospective Korean cohort observed for 6 years.

## 2. Materials and Methods

### 2.1. Study Design and Participants

The Korean Genome and Epidemiologic Survey-Health Examinees (KoGES-HEXA) cohort forms part of a large prospective cohort study to identify genetic and environmental factors of common complex diseases in Koreans, supported by government funding. The cohort comprises community residents and participants, men and women, aged ≥40 years at the baseline visit, who were recruited from the National Health Examinee Registry. These participants were recruited during the baseline survey conducted between 2004 and 2013 at 38 health examination centers and hospitals situated in eight Korean regions. The follow-up participants were then periodically invited to complete surveys by mail and telephone. For this analysis, the anonymized data of 173,195 participants ≥ 40 years were combined with the death certificate database of the National Statistical Office. The dataset of these participants consisted of anthropometric and clinical measurements, a lifestyle survey (i.e., diet, smoking, drinking, and physical activity), and a food frequency questionnaire (FFQ). Participants were enrolled in 2004 and followed up from 2012 to 2018. The study details have been published elsewhere [16].

**Figure 1:**
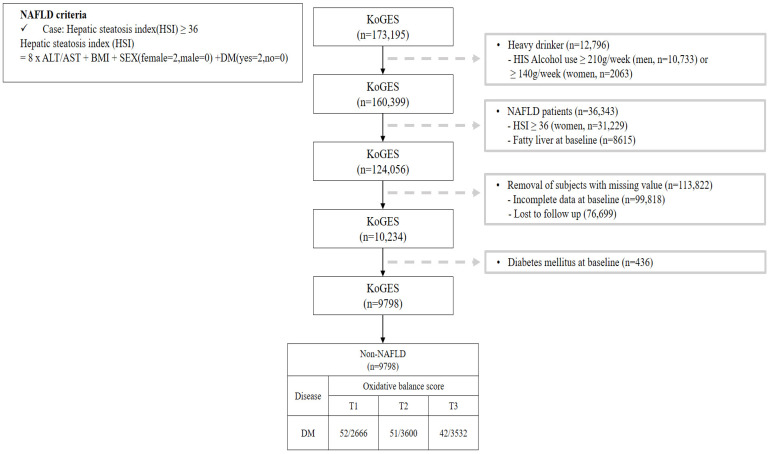
Flowchart for the selection of study participants.

This study investigated the OBS of patients without NAFLD and Figure 1 shows a flow chart describing this study. We excluded participants (*n* = 36,343) with a value for the hepatic steatosis index (HSI) [17] of over 36 among 173,195 participants. Additionally, participants with heavy alcohol use (≥210 g/week in men; 140 g/week in women) [18] (*n* = 12,796), lost to follow-up (*n* = 76,699), with missing covariates (*n* = 99,818), and with pre-existing diabetes (*n* = 436) were all excluded. After those exclusions, a total of 9798 participants without NAFLD were included with their disease history and mortality records.

Our study was performed in accordance with the Declaration of Helsinki and was approved by the Ethics Committee of the Korean Health and Genomic Study at the Korea National Institute of Health. The HEXA study protocol was reviewed and approved by the Institutional Review Board of the Korea Centers for Disease Control and Prevention (now the Korea Disease Control and Prevention Agency), and all study participants provided their written informed consent. Approval for this research was granted by the Institutional Review Board of Yongin Severance Hospital, with an assigned IRB number of 9-2023-0045.

### 2.2. Data Collection and Covariates

During the health examination, each participant completed a comprehensive questionnaire that captured information about their lifestyle and medical history and underwent a comprehensive medical examination provided by trained medical staff following standard procedures. Smoking status was classified as never smoker, former smoker, or current smoker. Bodyweight and height measurements were performed while participants were wearing light indoor clothing and no shoes, with a precision of 0.1 kg and 0.1 cm, respectively. Waist circumference (WC) was measured after normal exhalation midway between the lower rib margin and the iliac crest, parallel to the horizontal plane, to the nearest 0.1 cm. Body mass index (BMI) was calculated by dividing the weight by the height squared (kg/m^2^). Systolic and diastolic blood pressure (BP) were measured after a 10 min resting period in the seated position, using a standard mercury sphygmomanometer (Baumanometer; W.A. Baum Co. Inc., Copiague, NY, USA). Mean arterial pressure (MAP) was calculated as: (systolic BP + (2 × diastolic BP))/3. A “never smoker” was defined as a participant who had never smoked or had smoked fewer than 100 cigarettes in their lifetime. A “former smoker” was defined as a participant who had quit smoking and had smoked more than 100 cigarettes during their lifetime. Finally, a “current smoker” was defined as a participant who had smoked 100 cigarettes in their lifetime and who answered “currently smoking” in the questionnaire. Drinking status was defined as never drinker, light and moderate drinker (0–209 g/week in men; 0–139 g/week in women), or heavy drinker (≥210 g/week in men; ≥140 g/week in women), based on the frequency of alcohol consumption reported by the subjects [18]. Regular drinkers were defined as participants who drank alcohol at least once a month. Regular exercise was defined as regularly performing physical exercise that causes sweating. 

Hypertension was defined as SBP ≥ 140 mmHg, DBP ≥ 90 mmHg, or current use of hypertension medication [19]. Chronic kidney disease (CKD) was defined as an estimated glomerular filtration rate (eGFR) < 60 mL/min/1.73 m^2^. The eGFR was calculated using the CKD Epidemiology Collaboration (CKD-EPI) equation [20]. Dyslipidemia was defined as satisfying one of the definitions stated as follows: (1) hyper-low-density lipoprotein (LDL)-cholesterolemia was defined as serum LDL-cholesterol ≥ 160 mg/dL or taking lipid-lowering drugs; (2) hypo-high-density lipoprotein (HDL)-cholesterolemia was defined as serum HDL-cholesterol < 40 mg/dL; or (3) hypertriglyceridemia was defined as serum triglycerides ≥ 200 mg/dL [21].

A 103-item food frequency questionnaire (FFQ) was used to estimate dietary intake, and well-trained dietitians aided in the completion of the FFQ. The validity and reproducibility of the FFQ for the KoGES were previously demonstrated [22]. We calculated the daily total energy intake (kcal/day) as well as the dietary fiber (g/day), carotene (μg/day), riboflavin (mg/day), niacin (mg/day), vitamin B6 (mg/day), total folate (mcg/day), vitamin C (mg/day), vitamin E (ATE) (mg/day), calcium (mg/day), zinc (mg/day), total fat (g/day), and iron (mg/day) intake of the participants using the Computer Aided Nutritional Analysis program (CAN-pro version 5.0; The Korean Nutrition Society, Seoul, Republic of Korea).

Blood samples were collected from subjects via an antecubital vein following a 12 h overnight fast. The concentrations of total cholesterol, high-density lipoprotein (HDL) cholesterol, triglycerides, fasting plasma glucose (FPG), aspartate aminotransferase (AST), and alanine aminotransferase (ALT) were enzymatically measured using a Chemistry Analyzer (Hitachi 7600; Tokyo, Japan until August 2002; ADVIA 1650; Siemens, Tarrytown, NY, USA from September 2002). Further details of the study’s design and procedures have been described elsewhere [16].

### 2.3. Assessment of Oxidative Balance Scores

Table 1 shows the OBS components, including dietary and lifestyle factors that have antioxidant or pro-oxidant properties. The OBS was calculated as the sum of 11 antioxidant factors and 5 pro-oxidant factors selected based on previous studies [14,15,16,17,18,19].

Participants were categorized into sex-specific tertile groups based on the OBS (Table 1). Higher scores were given for increasing amounts of antioxidant factors (T1 scored 0, T2 scored 1, and T3 scored 2) and for decreasing amounts of pro-oxidants (T1 scored 2, T2 scored 1, and T3 scored 0).

Antioxidant factors included intakes of dietary fiber, carotene, riboflavin, niacin, vitamin B6, total folate, vitamin C, vitamin E (ATE), calcium, and zinc and physical activity. The scores for dietary fiber, carotene, riboflavin, niacin, vitamin B6, total folate, vitamin C, vitamin E (ATE), calcium, and zinc intake were assigned 0 through 2 points according to the sex-specific tertile values of each variable corresponding to high (score 2), intermediate (score 1), and low (score 0). Two points were given for very active physical activity, one for active physical activity, and 0 for inactive or low physical activity.

Pro-oxidant factors included total fat and iron intake, drinking status, smoking status, and obesity status. The scores for total fat and iron intake were assigned 0 through 2 points according to the sex-specific tertile values of each variable corresponding to low (score 2), intermediate (score 1), and high (score 0). For smoking status, the scores for never smoker, former smoker, and current smoker were 2, 1, and 0, respectively. For drinking status, the scores for a non-drinker and light and moderate drinker (0–29 g/day in men; 0–19 g/day in women) were 1 and 0, respectively. For obesity status, the scores for the low BMI group (≤23.23 kg/m^2^ in men; ≤22.26 kg/m^2^ in women), moderate BMI group (23.23–25.47 kg/m^2^ in men; 22.26–24.67 kg/m^2^ in women), and high BMI group (>25.47 kg/m^2^ in men; >24.67 kg/m^2^ in women) were 2, 1, and 0, respectively. The correlations between the OBS and each OBS component are shown in the Supplementary Materials, Figure S1. The OBS and nutrient factors (dietary fiber, carotene, riboflavin, niacin, vitamin B6, total folate, vitamin C, vitamin E (ATE), calcium, zinc, total fat, and iron) were negatively correlated except for total fat, and OBS and non-dietary factors (physical activity, alcohol consumption, BMI, and smoking status) were weakly correlated compared to nutrient factors. The sum of the OBS components ranged from 0 to 31 points.

### 2.4. Study Outcomes

Disease information was obtained through data linkage based on the personalized identification key code system. The HEXA cohort is linked to national data sources (Korea National Statistical Office), including mortality records. New-onset T2DM was defined using the diagnostic criteria of the American Diabetes Association Guidelines: (1) current use of glucose-lowering medications and insulin; (2) an 8 h FPG of 7.0 mmol/L (126 mg/dL) or greater; (3) an HbA1c level of 6.5% or greater; (4) or a plasma glucose (PG) level of 11.1 mmol/L (200 mg/dL) or greater 2 h after a 75 g oral glucose tolerance test (OGTT) [23]. Non-alcoholic fatty liver disease (NAFLD) was defined as a hepatic steatosis index score (HSI) ≥ 36, which was calculated using the following formula: HSI = 8 × ALT/AST + BMI + SEX (female = 2; male = 0) + DM (yes = 2; no = 0) [17].

### 2.5. Statistical Analysis

According to the basic level of the OBS, we divided the participants into three groups. Participants without NAFLD were divided according to OBS tertiles as follows: T1: ≤15, T2: 16–22, and T3: ≥23. All data are presented as the mean with standard deviation or percentage. According to the OBS tertiles, the baseline characteristics of this study’s population were compared using a Pearson’s chi-square test for categorical variables and an analysis of variance (ANOVA) model for continuous variables. Reverse Kaplan–Meier curves were used to assess the cumulative incidence of T2DM. The log-rank test was used to determine whether the distribution of cumulative T2DM incidence differed among groups. In multivariable analysis, after setting the lowest T2DM value tertile as the reference group, the hazard ratios (HRs) and 95% confidence intervals (CIs) for incident T2DM were calculated using the Cox proportional hazards regression model after adjusting for potential confounding variables. All analyses were performed using SAS version 9.2 software (SAS Institute Inc., Cary, NC, USA). All statistical tests were two-sided, and a *p*-value < 0.05 was considered to be statistically significant.

## 3. Results

Table 2 shows the baseline characteristics of this study’s population according to the OBS tertiles. A total of 2396 men and 7402 women were included in this study. The T1 group had higher levels or proportions of BMI, WC, total cholesterol, triglycerides, current smokers, current drinkers, dyslipidemia, and CKD and had lower levels or proportions of HDL-cholesterol and regular exercise. There were no significant differences between tertile groups for levels or proportions of systolic BP, diastolic BP, FPG, and hypertension.

Table 3 shows the relationship between the OBS and T2DM incidence among individuals without NAFLD. During the median 3.5-year follow-up period, a total of 145 participants developed new-onset T2DM. The incidence rates per 1000 person-years were 5.37 at T1, 4.00 at T2, and 3.45 at T3. Additionally, univariable analysis was used to determine whether each covariate influenced the incidence of T2DM (Table S1). After adjusting for age, sex, BMI, WC, systolic BP, diastolic BP, FPG, total cholesterol, HDL-cholesterol, triglycerides, smoking status, alcohol intake, regular exercise, hypertension, dyslipidemia, CKD, and total energy intake, the HRs (95% CIs) for new-onset T2DM were 0.79 (0.53–1.18) at T2 and 0.60 (0.39–0.93) at T3 (p for trend = 0.017), compared with referent T1.

**Figure 2:**
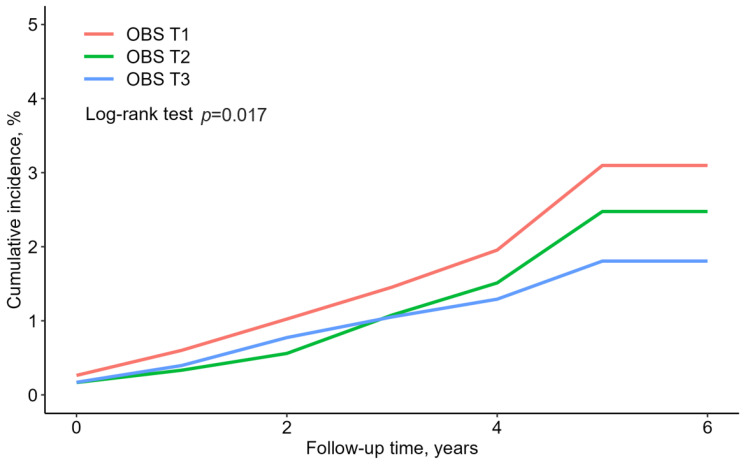
Reverse Kaplan–Meier curves of cumulative new-onset T2DM according to the OBS tertiles.

Figure 2 presents the rate of cumulative new-onset T2DM according to the OBS tertiles, presented as Kaplan–Meier curves. The T3 group showed the significantly lowest cumulative incident T2DM, followed by the T2 and T1 groups (log-rank test, *p* = 0.017).

Table 4 shows the results of the univariable analysis performed to determine the effect of each of the individual components of the OBS on T2DM incidence using subgroup analysis. For zinc, compared to the lowest first tertile, the HR (95% CI) of new-onset T2DM was significantly reduced in the T2 group, with 0.59 (0.38–0.89), but did not show a dose-dependent trend in the T3 group, with 0.82 (0.56–1.19). For BMI, the HRs (95% CIs) of new-onset T2DM with decreasing BMI were significantly reduced in the T2 group, with 0.61 (0.41–0.91), and in the T1 group, with 0.47 (0.31–0.71), with an inverse dose-dependent trend. For smoking, compared to current smokers, the HR (95% CI) of new-onset T2DM was significantly reduced in never smokers, with 0.42 (0.26–0.69), with an inverse dose-dependent trend. For dietary fiber, there was no significant trend in the univariable analysis, but after adjusting for age, sex, FPG, and total calorie intake in the multivariable analysis, there was a significant trend in the T2 group, with 0.55 (0.33–0.90), and the trend was maintained in the T3 group, with 0.56 (0.30–1.05), although it was not significant. Vitamin C showed a trend toward a decrease in T2DM incidence with increasing intake, but the trend was not statistically significant. The remaining components, such as carotene, riboflavin, niacin, vitamin B6, total folate, vitamin E, calcium, total fat, iron, physical activity, and alcohol consumption, showed no significant trends.

## 4. Discussion

In this study of a large community-based prospective cohort of Korean adults over a 6-year period, the incidence of T2DM in middle-aged and older adults without NAFLD was found to decrease with increasing OBS, even after adjusting for potential confounding variables. The T3 group had only 0.60 times the risk of developing T2DM compared to the T1 group, which was consistent with previous prospective cohort studies that have tracked the relationship between the OBS and T2DM incidence in the general population [8]. Our findings support the hypothesis that an appropriate balance of oxidant and antioxidant exposure is protective against T2DM even in populations without NAFLD.

There have been previous studies attempting to shed light on the relationship between the OBS and T2DM. A study of 476 Iranian patients with T2DM found that a higher OBS was associated with better glycemic control [9]. After adjusting for confounding factors, mean glycated hemoglobin and fasting glucose were significantly lower in the highest tertile of OBS compared to the lowest tertile [9]. However, this study was limited by the fact that it was a cross-sectional study and included patients who had already developed T2DM. Wu et al. used NHANES data to examine the cross-sectional relationship between the OBS and T2DM and found that the odds ratio was significantly lower in the highest quartile of OBS compared to the lowest quartile [10]. Kwon et al. showed that the OBS was inversely related to incident T2DM after adjusting for confounding variables in the general population through a KoGES prospective cohort study [8].

However, in the majority of patients with T2DM, other metabolic diseases are co-morbid, and there are numerous interactions between their pathogeneses. Although T2DM is generally described as the result of an imbalance between increased insulin resistance and decreased insulin secretion, the mechanisms are very complex and depend on a variety of contributing factors [24]. For example, cellular aging and dysfunction in various organs or tissues (e.g., adipose tissue, skeletal muscle, pancreas) contribute to the pathogenesis of T2DM as follows: (1) activation of pro-inflammatory pathways in both adipose tissue and skeletal muscle; (2) premature or accelerated biological aging induced by ectopic fat and central obesity-related factors and hyperglycemia; (3) pancreatic beta-cell aging and dysfunction; and (4) skeletal muscle loss and dysfunction [24]. These complex interactions can confound the impact of lifestyle modifications, such as those represented by the OBS, on incident T2DM [24].

The strong bidirectional relationship between T2DM and NAFLD is well recognized [11,12]. It is estimated that more than two-thirds of T2DM patients also have NAFLD [13]. NAFLD increases the risk of T2DM, and T2DM increases the risk of NAFLD progressing to liver fibrosis [25]. Mechanisms studied to date suggest that NAFLD may increase the risk of developing T2DM by exacerbating hepatic insulin resistance, leading to the release of inflammatory mediators and diabetic hepatokines such as fetuin-A/B and fibroblast growth factor 21 (FGF21) [26]. Although insulin resistance is speculated to be a major mechanism through which NAFLD and T2DM increase each other’s risk, it remains largely unclear whether insulin resistance is actually a cause or effect of NAFLD and T2DM, as well as its overall relationship with other metabolic diseases [15]. Given the close relationship between the two diseases, NAFLD potentially interferes with the impact of lifestyle modification represented by the OBS on T2DM incidence. In this study, by studying a population without NAFLD [15], we sought to maximize our understanding of the interdependence of these factors in T2DM incidence [14].

One method for clinically understanding the impact of oxidative stress on many chronic diseases is the OBS, which has undergone many iterations since its first publication in 2002 [27]. The OBS is a composite measure that includes dietary and lifestyle factors, with a higher OBS indicating that antioxidants predominate over oxidants. Based on previous research on the relationship between the OBS and chronic disease, we included the most studied factors, such as: vitamin C, vitamin E, beta-carotene, total iron, alcohol consumption, and smoking status [10,28]. However, some components, such as lycopene and lutein, were not included in the KoGES dataset and were excluded from the analysis. In this study, although the OBS was limited to exogenous exposures, including dietary and non-dietary lifestyle factors, several studies have demonstrated that the OBS is associated with biomarkers of oxidative stress, such as C-reactive protein (CRP) [29] and γ-glutamyl transferase (GGT) levels [30]. Therefore, the OBS is sufficiently valid for the assessment of oxidative balance. To our knowledge, no study has identified strong evidence of an epidemiologic inverse relationship between the OBS and T2DM risk in a prospective cohort without NAFLD. Therefore, we investigated the effect of the OBS on the incidence of T2DM in middle-aged and older adults without NAFLD through a large community-based prospective Korean cohort observed for 6 years.

In this study, when univariable analysis was performed on non-dietary OBS factors, the incidence of T2DM was clearly lower in the never smokers than in former and current smokers, a result that was consistent with previous studies analyzing the impact of the OBS on chronic disease [8,31]. By negatively affecting pancreatic beta cell function and insulin sensitivity, promoting inflammation, and contributing to increased visceral fat, smoking significantly increases the incidence of clinical diabetes [32]. The higher BMI group (T3) also had a significantly higher incidence of T2DM than the T2 and T1 BMI groups. This was consistent with previous findings that obesity affects the incidence of T2DM by increasing insulin resistance through a variety of mechanisms [33].

In the univariable analysis related to dietary OBS factors, zinc was significant at T2 but not at T3, and this remained true in the multivariable analysis corrected for age and sex as confounders. There is evidence that zinc is essential for the proper processing of insulin by beta cells (formation of insulin hexamers), as well as storage and secretion [34]; thus, zinc has a preventative effect on metabolic diseases associated with insulin resistance, including diabetes [34,35]. However, in clinical studies to date, metabolic improvements have been observed in the majority of type 2 diabetic patients in studies that reported Zn deficiency [36], whereas no improvements in fasting blood glucose levels or HbA1c were observed in trials that did not report Zn deficiency [36]. The recommended dietary allowance (RDA) for zinc is 11 mg/day for men and 8 mg/day for women, and the upper limit of tolerance (UL) defined in the 2001 Dietary Reference Intakes report issued by the US Institute of Medicine/Food and Nutrition board is 40 mg/day for adults [36,37]. In our study, the cut-off for the highest zinc intake (T3) was >8.05 mg in men and >7.44 mg in women, which may have included some participants who, whilst not exceeding the upper limit of tolerable intake, exceeded the recommended daily intake. This may be one of the reasons why the protective effect against T2DM was significant at T2 but disappeared at T3.

Furthermore, given the interactive mechanisms suggested by previous studies, it may be more important to consider the OBS as a whole than to consider individual components alone. A healthy dietary pattern with high intakes of fruits, vegetables, nuts, and fish, which are rich sources of vitamins, minerals, polyphenols, and healthy fats, is associated with increased insulin sensitivity and reduced inflammation [38]. Physical activity has a variety of positive effects, including improving serum lipids, enhancing peripheral insulin sensitivity, reducing blood pressure, reducing inflammation, and promoting weight loss [39,40]. A healthy lifestyle, including a combination of healthy eating, physical activity, smoking cessation, and a healthy weight, as well as education about all of the aforementioned factors, is strongly associated with a lower risk of T2DM [41,42]. This is very similar to the dietary and lifestyle recommendations in the American Gastroenterological Association’s clinical guidelines for NAFLD patients [43]. Importantly, this study provides some clinical support for the possibility that these recommendations related to oxidative balance may play a role in mechanisms independent of NAFLD.

There are several limitations to this study. First, follow-up information on nutrient intake is not specifically available in the KoGES dataset, so only baseline OBS and confounders were used in the analysis. Further studies will need to take this into account, as changes in the OBS over time due to disease progression and lifestyle changes may have an additional impact on the development of T2DM. Second, although each OBS component has different antioxidant or pro-oxidant properties, which may have different effects on the development of T2DM, all OBS components were equally weighted. Therefore, the contribution of each component may be inaccurately reflected, and further studies are needed to clarify this. However, previous studies have not found significant differences between unweighted and weighted OBS components in their associations with various cancers and mortality [44,45]. Third, the OBS only included dietary and non-dietary lifestyle exposures. The data obtained from KoGES did not include information on endogenous factors such as individual pro- and anti-inflammatory cytokines and their associated genes that contribute to oxidative balance. Fourth, this study was conducted using the KoGES-HEXA cohort (n = 173,195), which has a larger number of participants than the KoGES Ansan and Ansung cohorts (n = 10,030) used in previous studies; however, there was only one follow-up, and this may have led to an inaccurate timing of DM diagnosis compared to the Ansan and Ansung cohorts, which employed biannual follow-ups. In addition, due to the short mean follow-up period of 3.5 years, there were fewer new-onset T2DM cases than in previous studies, which may have reduced the statistical power. Nevertheless, we found significant results for the impact of the OBS on T2DM morbidity. Finally, NAFLD was defined using the hepatic steatosis index (HSI), a validated non-invasive method, instead of histologic information from liver ultrasound imaging or a liver biopsy. However, it should be noted that, although a liver biopsy is the gold standard for diagnosing NAFLD, it is not frequently used due to its invasiveness, high cost, and complications [46]. Despite the above limitations, this study has the clear strength of identifying the impact of dietary and non-dietary lifestyle factors on T2DM in a large population-based prospective cohort over a 6-year period, in a population for which the confounder of NAFLD was clearly excluded.

In conclusion, the OBS is predicted to be a trustworthy indicator of new-onset T2DM in middle-aged and older population without NAFLD, unaffected by alcohol consumption. Our findings suggest that the OBS could be an additional valuable tool for assessing incident T2DM among individuals without NAFLD.

## Figures and Tables

**Table 1:** Components of the oxidative balance score.

OBS Components	Property	Male			Female		
		T1	T2	T3	T1	T2	T3
Dietary OBS components							
Dietary fiber (g/day)	A	≤5.65	5.65< ≤7.85	>7.85	≤5.35	5.35< ≤7.7	>7.7
Carotene (ug/day)	A	≤2627.76	2627.76< ≤4735.96	>4735.96	≤2744.36	2744.36< ≤4883.75	>4883.75
Riboflavin (mg/day)	A	≤0.84	0.84< ≤1.16	>1.16	≤0.8	0.8< ≤1.11	>1.11
Niacin (mg/day)	A	≤12.43	12.43< ≤16.76	>16.76	≤11	11< ≤14.88	>14.88
Vitamin B_6_ (mg/day)	A	≤1.28	1.28< ≤1.69	>1.69	≤1.18	1.18< ≤1.6	>1.6
Total folate (mcg/day)	A	≤264.07	264.07< ≤371.51	>371.51	≤247.1	247.1< ≤354.44	>354.44
Vitamin C (mg/day)	A	≤65.53	65.53< ≤104.21	>104.21	≤67.89	67.89< ≤108.89	>108.89
Vitamin E (ATE) (mg/day)	A	≤6.99	6.99< ≤9.75	>9.75	≤6.69	6.69< ≤9.58	>9.58
Calcium (mg/day)	A	≤367.08	367.08< ≤528.09	>528.09	≤343.99	343.99< ≤504.23	>504.23
Zinc (mg/day)	A	≤6.38	6.38< ≤8.05	>8.05	≤5.7	5.7< ≤7.44	>7.44
Total fat (g/day)	P	≤25.52	25.52< ≤38.31	>38.31	≤22.92	22.92< ≤34.57	>34.57
Iron (mg/day)	P	≤10.77	10.77< ≤14.69	>14.69	≤10.08	10.08< ≤14.15	>14.15
Lifestyle OBS components							
Physical activity ^a^	A	Inactive or low activity	Active	Very active	Inactive or low activity	Active	Very active
Alcohol (g/day) ^b^	P	None	0< ≤30	≥30	None	0< ≤20	≥20
Body mass index (kg/m^2^)	P	≤23.23	23.23< ≤25.47	>25.47	≤22.26	22.26< ≤24.67	>24.67
Smoking status ^c^	P	Never smoker	Former smoker	Current smoker	Never smoker	Former smoker	Current smoker
Tertile Scores for “A”		T1 score: 0	T2 score: 1	T3 score: 2	T1 score: 0	T2 score: 1	T3 score: 2
Tertile Scores for “P” ^d^		T1 score: 2	T2 score: 1	T3 score: 0	T1 score: 2	T2 score: 1	T3 score: 0

Low, intermediate, and high categories correspond to sex-specific tertile values among participants in the KoGES at the baseline survey. Abbreviations: OBS, oxidative balance score; A, antioxidant; P, pro-oxidant; ATE, alpha-tocopherol equivalents; KoGES, Korean Genome and Epidemiology Study. ^a^ Inactive, limited physical activity, such as hospitalized; Low activity, spending most of their time in sedentary activities, such as general office workers who do not actively exercise regularly during their free time; Active, mainly sedentary but does standing work, commuting, shopping, housework, regular light exercise; Very active, mainly engaged in standing work or engaged in active leisure activities such as exercise. ^b^ Never smoker, participants who had never smoked or had smoked <100 cigarettes in their lifetime; Former smoker, participants who had quit smoking and had smoked ≥100 cigarettes in their lifetime; Current smoker, participants who had smoked >100 cigarettes in their lifetime and answered “currently smoking”. ^c^ Never drinker, participants who answered “None” in the questionnaire; Light and moderate drinker, 0–209 g/week in men, 0–139 g/week in women; Heavy drinker, ≥210 g/week in men, ≥140 g/week in women. ^d^ For alcohol, T1 scored 1, T2 scored 0, and no T3 score for both men and women.

**Table 2:** Baseline characteristics of study population by oxidative balance score tertiles.

	Total	Group 1	Group 2	Group 3	
	(*n* = 9798)	T1 [3, 15] (*n* = 2666)	T2 (15, 22] (*n* = 3600)	T3 (22, 31] (*n* = 3532)	*p*-Value
Sex (men)	2396 (24.5)	769 (28.8)	887 (24.6)	740 (21.0)	<0.001
Age (years)	54.0 ± 8.0	53.5 ± 8.1	53.8 ± 8.2	54.5 ± 7.7	<0.001
BMI (kg/m^2^)	22.8 ± 2.2	23.2 ± 2.3	22.8 ± 2.2	22.5 ± 2.2	<0.001
Waist circumference (cm)	77.4 ± 7.6	78.4 ± 7.7	77.5 ± 7.6	76.7 ± 7.4	<0.001
Systolic BP (mmHg)	120.5 ± 14.4	120.7 ± 14.5	120.2 ± 14.5	120.7 ± 14.4	0.284
Diastolic BP (mmHg)	73.9 ± 9.2	74.2 ± 9.4	73.8 ± 9.2	73.7 ± 9.1	0.065
FPG (mg/dL)	92.4 ± 12.0	92.4 ± 12.4	92.3 ± 11.4	92.5 ± 12.3	0.663
Total cholesterol (mg/dL)	199.0 ± 35.0	200.4 ± 35.7	198.2 ± 34.7	198.8 ± 34.8	0.042
HDL cholesterol (mg/dL)	56.8 ± 13.7	56.3 ± 13.6	56.7 ± 13.7	57.3 ± 13.8	0.008
Triglyceride (mg/dL)	111.7 ± 68.3	115.3 ± 68.5	111.8 ± 68.1	109.0 ± 68.2	0.001
Smoking status, *n* (%) ^a^					<0.001
Never smoker	7958 (81.2)	2027 (76.0)	2919 (81.1)	3012 (85.3)	
Former smoker	1224 (12.5)	382 (14.3)	453 (12.6)	389 (11.0)	
Current smoker	616 (6.3)	257 (9.6)	228 (6.3)	131 (3.7)	
Alcohol intake, *n* (%) ^b^					<0.001
Never drinker	5763 (58.8)	1369 (51.4)	2083 (57.9)	2311 (65.4)	
Former drinker	310 (3.2)	84 (3.2)	117 (3.2)	109 (3.1)	
Current drinker	3725 (38.0)	1213 (45.5)	1400 (38.9)	1112 (31.5)	
Regular exercise (Yes) ^c^	5604 (57.2)	1339 (50.2)	2051 (57.0)	2214 (62.7)	<0.001
Hypertension, *n* (%)	1526 (15.6)	421 (15.8)	547 (15.2)	558 (15.8)	0.731
Dyslipidemia, *n* (%)	2507 (25.6)	739 (27.7)	909 (25.2)	859 (24.3)	0.008
CKD, *n* (%)	73 (0.7)	17 (0.6)	37 (1.0)	19 (0.5)	0.042

Data are expressed as the mean (SD), median (IQR), or percentage. Abbreviations: BMI, body mass index; BP, blood pressure; FPG, fasting plasma glucose; HDL, high-density lipoprotein; CKD, chronic kidney disease; eGFR, estimated glomerular filtration rate; LDL, low-density lipoprotein. *p*-Values were calculated with the use of ANOVA test or chi-square test. ^a^ Never smoker, participants who had never smoked or had smoked <100 cigarettes in their lifetime; Former smoker, participants who had quit smoking and had smoked ≥100 cigarettes in their lifetime; Current smoker, participants who had smoked >100 cigarettes in their lifetime and answered “currently smoking”. ^b^ Never drinker, participants who answered “None” in the questionnaire; Light and moderate drinker, 0–209 g/week in men, 0–139 g/week in women; Heavy drinker, ≥210 g/week in men, ≥140 g/week in women. ^c^ Regularly performing exercise that causes sweating. Hypertension, SBP ≥ 140 mmHg, DBP ≥ 90 mmHg, or current use of hypertensive medication; CKD, eGFR < 60 mL/min/1.73 m^2^; Dyslipidemia, satisfying one of the following criteria: (1) serum LDL-cholesterol ≥ 160 mg/dL or taking lipid-lowering drug; (2) serum HDL-cholesterol < 40 mg/dL; (3) serum triglycerides ≥ 200 mg/dL.

**Table 3:** Hazard ratios and 95% confidence intervals for incident type 2 diabetes by oxidative balance score.

		Group 1	Group 2	Group 3	
		T1 [3, 15] (*n* = 2666)	T2 (15, 22] (*n* = 3600)	T3 (22, 31] (*n* = 3532)	*p* for Trend
New cases of type 2 diabetes mellitus, *n*		52	51	42	
Mean follow-up, years		3.63	3.54	3.44	
Person-years of follow-up		9682	12,736	12,157	
Incidence rate/1000 person-years		5.37	4.00	3.45	
Incidence rate per 1000 person-years					
Model 1	HR (95% CI)	1.00 (reference)	0.76 (0.52–1.12)	0.66 (0.44–0.99)	0.043
	*p*-value		0.164	0.044	
Model 2	HR (95% CI)	1.00 (reference)	0.78 (0.52–1.16)	0.62 (0.40–0.95)	0.029
	*p*-value	-	0.213	0.029	
Model 3	HR (95% CI)	1.00 (reference)	0.79 (0.53–1.18)	0.60 (0.39–0.93)	0.017
	*p*-value	-	0.247	0.023	

Model 1: adjusted for age and sex. Model 2: adjusted for age, sex, BMI, WC, systolic BP, diastolic BP, FPG, total cholesterol, HDL-cholesterol, and triglycerides. Model 3: adjusted for age, sex, BMI, WC, systolic BP, diastolic BP, FPG, total cholesterol, HDL-cholesterol, triglycerides, smoking status, alcohol intake, regular exercise, hypertension, dyslipidemia, CKD, and total energy intake. Abbreviations: BMI, body mass index; BP, blood pressure; FPG, fasting plasma glucose; HDL, high density lipoprotein.

**Table 4:** Hazard ratios and 95% confidence intervals from univariable and multivariable analyses of the incidence of type 2 diabetes according to components of the oxidative balance score.

	Univariable		Multivariable *	
	HR (95% CI)	*p*-Value	HR (95% CI)	*p*-Value
Dietary OBS components				
Dietary fiber (ref. T1)				
T2	0.76 (0.51–1.13)	0.182	0.55 (0.33–0.90)	0.017
T3	0.74 (0.50–1.10)	0.132	0.56 (0.30–1.05)	0.071
Carotene (ref. T1)				
T2	0.85 (0.56–1.29)	0.437	1.01 (0.62–1.64)	0.982
T3	1.17 (0.79–1.73)	0.425	2.02 (1.18–3.44)	0.010
Riboflavin (ref. T1)				
T2	0.93 (0.63–1.38)	0.721	1.24 (0.76–2.03)	0.395
T3	0.81 (0.54–1.21)	0.301	0.91 (0.48–1.71)	0.766
Niacin (ref. T1)				
T2	0.85 (0.58–1.26)	0.429	0.79 (0.48–1.29)	0.338
T3	0.76 (0.51–1.13)	0.180	0.74 (0.39–1.39)	0.347
Vitamin B6 (ref. T1)				
T2	0.85 (0.58–1.26)	0.429	1.79 (1.08–2.96)	0.024
T3	0.76 (0.51–1.13)	0.180	0.84 (0.42–1.69)	0.626
Total folate (ref. T1)				
T2	1.29 (0.88–1.90)	0.188	0.57 (0.35–0.93)	0.025
T3	0.71 (0.46–1.10)	0.125	0.65 (0.36–1.19)	0.161
Vitamin C (ref. T1)				
T2	0.68 (0.45–1.01)	0.055	1.15 (0.70–1.88)	0.583
T3	0.71 (0.48–1.04)	0.080	1.15 (0.65–2.06)	0.626
Vitamin E (ATE) (ref. T1)				
T2	0.85 (0.57–1.29)	0.454	1.28 (0.80–2.05)	0.309
T3	0.94 (0.63–1.39)	0.758	1.12 (0.64–1.98)	0.693
Calcium (ref. T1)				
T2	0.86 (0.58–1.28)	0.464	0.71 (0.44–1.15)	0.163
T3	0.84 (0.56–1.25)	0.383	0.98 (0.59–1.62)	0.927
Zinc (ref. T1)				
T2	0.59 (0.38–0.89)	0.013	0.67 (0.40–1.12)	0.124
T3	0.82 (0.56–1.19)	0.289	0.96 (0.51–1.80)	0.906
Total fat (ref. T3)				
T2	1.08 (0.72–1.61)	0.723	0.75 (0.47–1.20)	0.228
T1	1.13 (0.76–1.69)	0.540	0.50 (0.27–0.90)	0.020
Iron (ref. T3)				
T2	1.11 (0.75–1.64)	0.611	1.17 (0.71–1.93)	0.542
T1	1.11 (0.74–1.67)	0.602	1.08 (0.55–2.12)	0.829
Lifestyle OBS components				
Physical activity (ref. inactive or low activity) ^a^				
Active	1.34 (0.80–2.22)	0.264	1.20 (0.71–2.00)	0.497
Very active	0.92 (0.41–2.07)	0.841	1.02 (0.45–2.30)	0.963
Alcohol (ref. Male: 0–30 g/day; Female: 0-20 g/day)				
None	1.18 (0.84–1.66)	0.346	1.38 (0.94–2.03)	0.100
Body mass index (ref. T3)				
T2	0.61 (0.41–0.91)	0.016	0.73 (0.49–1.10)	0.137
T1	0.47 (0.31–0.71)	<0.001	0.52 (0.33–0.80)	0.003
Smoke (ref. Current smoker) ^b^				
Former smoker	0.64 (0.35–1.17)	0.145	1.23 (0.58–2.61)	0.586
Never smoker	0.42 (0.26–0.69)	<0.000	1.39 (0.63–3.09)	0.412

* Adjusted for age, sex, FPG, and total calorie intake. Abbreviations: OBS, oxidative balance score; ATE, alpha-tocopherol equivalents; FPG, fasting plasma glucose. ^a^ Inactive, limited physical activity, such as hospitalized; Low physical activity, spending most of their time in sedentary activities, such as general office workers who do not actively exercise regularly during their free time; Active, mainly sedentary but does standing work, commuting, shopping, housework, regular light exercise; Very active, mainly engaged in standing work or engaged in active leisure activities such as exercise. ^b^ Never smoker, participants who had never smoked or had smoked <100 cigarettes in their lifetime; Former smoker, participants who had quit smoking and had smoked ≥100 cigarettes in their lifetime; Current smoker, participants who had smoked >100 cigarettes in their lifetime and answered “currently smoking”.

## Data Availability

The datasets used and analyzed in the current study are available from the corresponding author upon reasonable request.

## Supplementary Materials

The following supporting information can be downloaded at: https://www.mdpi.com/article/10.3390/antiox13010107/s1, Figure S1: Correlations between the OBS and the OBS components; Table S1: Hazard ratios and 95% confidence intervals from univariable and multivariable (model 1–3) analyses of the type 2 diabetes inci-dence by oxidative balance score and variables of baseline characteristics.
