# Supplementary material for: Oxidative Balance Score and New-Onset Type 2 Diabetes Mellitus in Korean Adults without Non-Alcoholic Fatty Liver Disease: Korean Genome and Epidemiology Study-Health Examinees (KoGES-HEXA) Cohort
Source: Antioxidants (Basel). 2024 Jan 16;13(1):107. doi: 10.3390/antiox13010107 (PMC10812514; doi:10.3390/antiox13010107)
Supplement: Supplementary file 1 [file antioxidants-13-00107-s001.zip › antioxidants-2750001-supplementary.pdf]

Supplementary Materials

Supplementary Table S1. Hazard ratios and 95% confidence intervals from univariable and multivariable (model 1-3) analyses of the type 2 diabetes incidence by oxidative balance score and variables of baseline characteristics.

Variables	Univariable		Model 1		Model 2		Model 3	
	HR (95% CI)	p value	HR (95% CI)	p value	HR (95% CI)	p value	HR (95% CI)	p value
OBS								
Group 1	reference		reference		reference		reference	
Group 2	0.78 (0.53-1.15)	0.211	0.76 (0.52-1.12)	0.164	0.78 (0.52-1.16)	0.213	0.79 (0.53-1.18)	0.247
Group 3	0.66 (0.44-0.99)	0.046	0.66 (0.44-0.99)	0.044	0.62 (0.40-0.95)	0.029	0.60 (0.39-0.93)	0.023
Men	1.95 (1.40-2.73)	<.001	1.55 (1.10-2.19)	0.012	0.75 (0.50-1.15)	0.185	0.93 (0.51-1.68)	0.806
Age (year)	1.06 (1.04-1.09)	<.001	1.06 (1.04-1.08)	<0.001	1.05 (1.03-1.07)	<0.001	1.03 (1.01-1.06)	0.009
BMI (kg/m2)	1.19 (1.11-1.28)	<.001			1.07 (0.96-1.19)	0.222	1.06 (0.95-1.19)	0.270
WC (cm)	1.07 (1.05-1.09)	<.001			1.02 (0.98-1.05)	0.354	1.02 (0.99-1.06)	0.245
Systolic BP (mmHg)	1.03 (1.02-1.04)	<.001			1.02 (1.00-1.04)	0.021	1.02 (1.00-1.04)	0.069
Diastolic BP (mmHg)	1.04 (1.02-1.05)	<.001			1.00 (0.97-1.03)	0.938	1.00 (0.98-1.03)	0.753
FPG (mg/dl)	1.03 (1.03-1.04)	<.001			1.03 (1.03-1.04)	<0.001	1.03 (1.03-1.04)	<0.001
Total cholesterol (mg/dl)	1.00 (0.99-1.00)	0.838			0.99 (0.99-1.00)	0.047	0.99 (0.99-1.00)	0.003
HDL cholesterol (mg/dl)	0.97 (0.95-0.98)	<.001			0.99 (0.98-1.01)	0.513	1.01 (0.99-1.03)	0.321
Triglyceride (mg/dl)	1.00 (1.00-1.00)	<.001			1.00 (1.00-1.00)	0.084	1.00 (1.00-1.00)	0.372
Smoking status [a]								
Never smoker	reference						reference	
Former smoker	1.52 (0.98-2.37)	0.064					0.72 (0.38-1.39)	0.331
Current smoker	2.38 (1.46-3.89)	<.001					0.71 (0.32-1.56)	0.392
Alcohol intake [b]								
Never drinker	reference						reference	
Former drinker	2.67 (1.46-4.88)	0.001					2.10 (1.07-4.14)	0.031
Current drinker	0.86 (0.60-1.22)	0.396					0.76 (0.51-1.14)	0.179
Regular exercise [c]	1.02 (0.73-1.42)	0.908					1.23 (0.87-1.76)	0.245
Hypertension	3.13 (2.23-4.39)	<.001					1.35 (0.92-1.99)	0.127
Dyslipidemia	2.65 (1.91-3.67)	<.001					2.26 (1.45-3.50)	<0.001
CKD	2.93 (0.94-9.21)	0.065					2.05 (0.64-6.55)	0.225

Total energy intake	0.88 (0.57-1.35)	0.550			1.06 (0.60-1.90)	0.836

Data are expressed as the mean (SD), median (IQR) or percentage.

Abbreviations: OBS, oxidative balance score; BMI, Body mass index; WC, Waist circumference; BP, blood pressure; FPG, fasting plasma glucose; HDL, high density lipoprotein; CKD, chronic kidney disease; eGFR, estimated glomerular filtration rate; LDL, low-density lipoprotein.

p values were calculated with the use of ANOVA-test or chi-square test.

[a] Never smoker, participants who had never smoked or had smoked < 100 cigarettes in their lifetime; Former smoker, participants who had quit smoking and had smoked ≥100 cigarettes in their lifetime; Current smoker, participants who had smoked > 100 cigarettes in their lifetime and answered "currently smoking".

[b] Never drinker, participants who answered “None” in the questionnaire; Light and moderate drinker, 0–209 g/week in men, 0–139 g/week in women; Heavy drinker, ≥210 g/week in men, ≥140 g/week in women.

[c] Regularly performing an exercise that causes sweating.

Hypertension, SBP ≥ 140 mmHg or DBP ≥ 90 mmHg, or current use of hypertensive medication; CKD, eGFR < 60 mL/min/1.73 m2; Dyslipidemia, satisfying one of the following criteria: (1) serum LDL-cholesterol ≥ 160 mg/dL or taking lipid-lowering drug; (2) serum HDL-cholesterol < 40 mg/dL; (3) serum triglyceride ≥ 200 mg/dL.

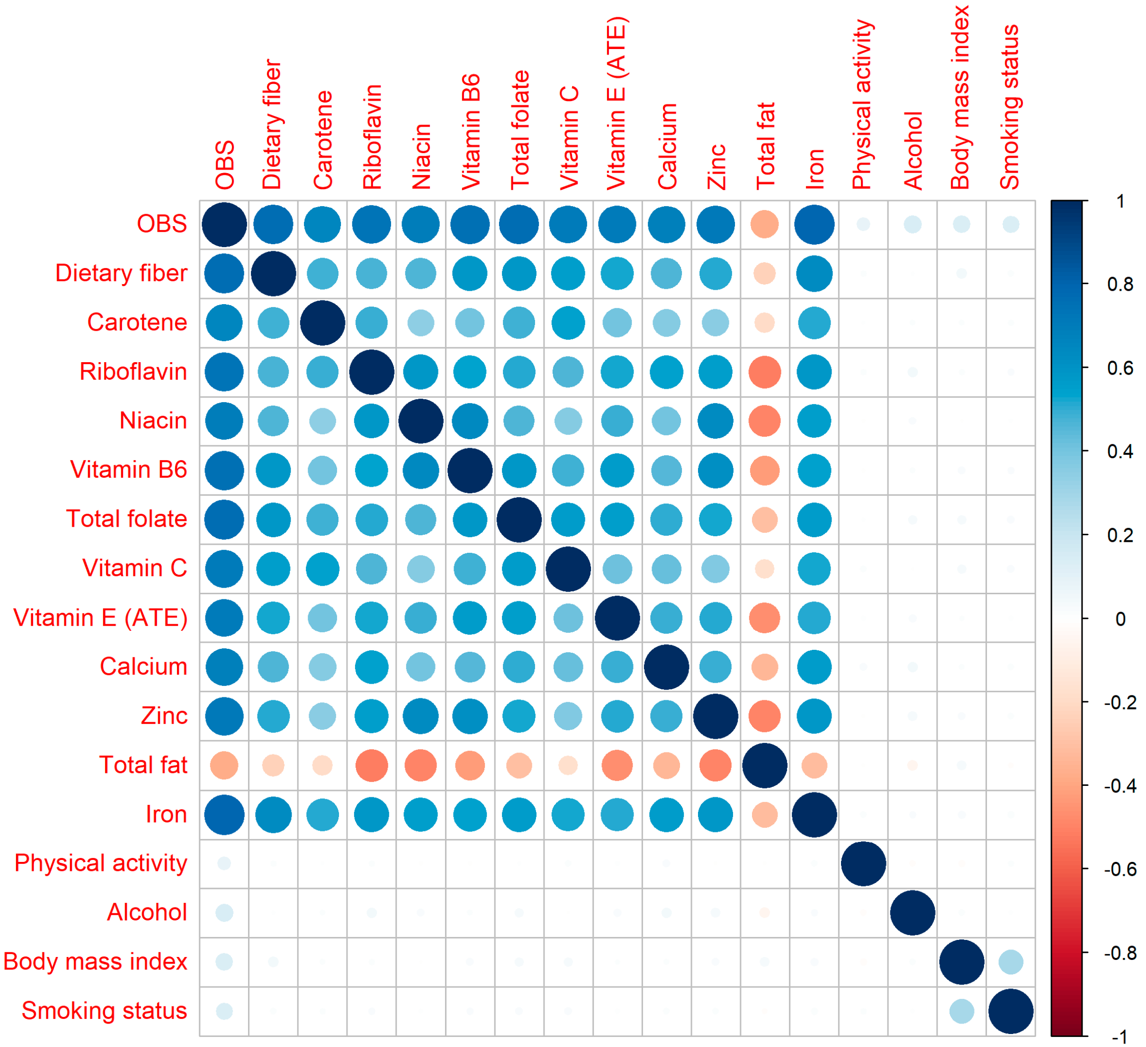

Supplementary Figure S1. Correlations between the OBS and the OBS components. The color values of the cells are scaled cells are scaled from red (negative correlation) to blue (positive correlation), proportional to the strength of the association, as indicated in the color scale (at the right side of the panel). Abbreviations: OBS, oxidative balance score; ATE, alpha-tocopherol equivalents.
